# Contact analysis and strength calculations of involute spline couplings

**DOI:** 10.1038/s41598-023-27615-2

**Published:** 2023-01-07

**Authors:** Shuting Li

**Affiliations:** Department of Mechanical, Electrical and Electronic Engineering, Shimane University, 1060 Nishikawatsu-Cho, Matsue, 690-8504 Japan

**Keywords:** Mechanical engineering, Mathematics and computing

## Abstract

A finite element method (FEM) is presented to conduct loaded tooth contact analysis and strength calculations of involute spline couplings. Special FEM software has been developed. Contact, bending and shear stresses of the spline couplings are analyzed by the developed software and they are compared with results obtained by an approximation method. It is found that “Edge loads” exist at tooth tip and root contacts and also exist at two sides of the face width when the pair of teeth has different face widths. Effects of tooth profile deviations and pitch errors on tooth contacts are also investigated by the developed software respectively. It is found that tooth contact and stresses are affected by the tooth profile deviations and pitch errors greatly. Finally, reliability of the presented method and software are discussed.

## Introduction

Involute spline couplings are often used in cars, robots, and other machines to connect two shafts and transmit a great torque. Though the spline couplings are used in machine design very early, strength calculation problem of this kind of couplings has not been solved so far. Designers must use an approximation method to conduct strength calculations of the spline couplings at the present situation.

Hayashi^1^ investigated torsional stiffness and a yield torque which was determined by the maximum shear stress produced by a loaded torque of spline couplings in theory. The torsional stiffness of the couplings was calculated theoretically, and the results were compared with the measured ones. But contact pressure on tooth surfaces, bending and shear stresses at tooth root could not be investigated. Burgtorf^2^ introduced fatigue failure patterns of spline couplings and explained reasons to resulted in the fatigue failures. It was pointed out that the cumulative pitch errors of the teeth were a substantial factor to affect loading-capacity of the spline couplings. Yeung^3^ studied stress calculations of the spline couplings with a taper using the boundary element method. Calculation results showed that the maximum principal stress in the shaft was reduced about 14% when an optimal tapering of 0.54° was added to the spline couplings. Schäfer^4^ introduced some industrial applications of modified splines to increase loading-capacity of the spline couplings through changing design parameters. Chase^5^ studied stress calculation methods of the spline couplings two dimensionally using an analytical method by suggesting a new, sequential model for spline tooth contact. Calculation results were compared ones obtained by commercial software. Pardhi1^6^ and Patil^10^ conducted stress analyses of spline couplings using ANSYS software. Shear stresses at the tooth root were analyzed and compared with an experimental result obtained by a photo elasticity. CURÀ^7^ also analyzed contact pressure on the tooth surfaces of the spline couplings using a two-dimensional (2D), FEM. Tjernberg^8^ investigated load distribution of spline teeth using an analytical method. Curàa^9^ conducted a contact analysis of the spline couplings using Commercial software and tooth contact stresses were investigated. Since FEM models used in this paper were very rough, it was necessary to confirm calculation results with an experiment. Also, tooth bending strength and shear strength could not be investigated in the paper.

Though there are so many papers available on the spline couplings, contact pressure distributions on the surfaces of contact teeth have not been analyzed successfully and effects of tooth profile deviations and pitch errors on tooth contacts have not been investigated. Especially, “Edge load” phenomenon of the contact teeth has not been analyzed. So, the reality is that strength calculation problem of the spline couplings has not been solved so far.

This paper presents a new FEM to conduct loaded tooth contact analysis and stress calculations of the involute spline couplings. This new FEM has many advantages that commercial software cannot have. For examples, this new FEM can save a lot of computer memories in the contact analysis. So, it can solve all-tooth-contact problem of the spline couplings very precisely and quickly even if number of teeth of the spline becomes very great. But the commercial software cannot solve this problem because of computer memory limit. Also, tooth profile deviations and pitch errors of the spline teeth can be considered very easily and precisely in the contact analysis when the new FEM is used. But it shall be very difficult to do the same analyses if the commercial software is used. Special software is also developed according to the principle of the new FEM.

## Involute spline couplings used as research objects

**Figure 1:**
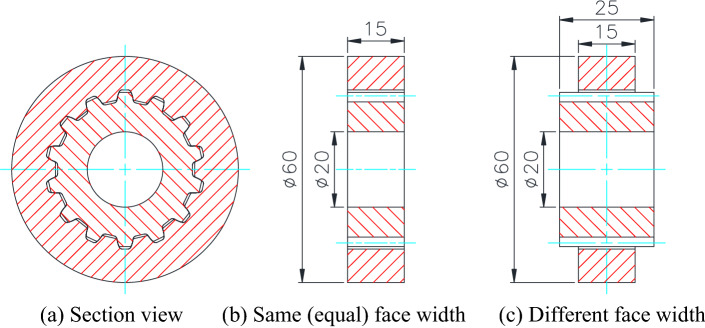
Structures of the involute spline couplings used as research objects.

Gearing parameters of the involute spline couplings used as research objects in this paper are given in Table 1. Figure 1a–c are section views of the spline couplings. Figure 1b is a case that the internal spline and the external spline have the same (equal) face widths. Figure 1c is a case that the internal spline and the external spline have different face widths. Figure 1c is used to investigate the effect of different face widths on tooth contact, bending and shear stresses of the spline couplings.

**Table 1:** Gearing parameters of the involute spline couplings used in this paper.

Items	Symbol	Results
Tooth profile		Involute
Number of teeth	$Z$	14
Module	$m$	2.5 (mm)
Pressure angle	${\alpha }_{c}$	20 (degrees)
Shifting coefficient	$x$	+ 0.8
Edge radius of cutter	${r}_{c}$	0.375 m
Addendum		0.4$m$ (mm)
Clearance		0.25$m$ (mm)
Face width of the hub	$b$	20 (mm)
Diameter of the meshing pitch circle	${D}_{m}$	${D}_{m}=(Z+2x)m$
Loaded torque	${T}_{z}$	411.6 (Nm)

## The principle used for contact analysis of the spline couplings

### The principle of contact analysis

**Figure 2:**
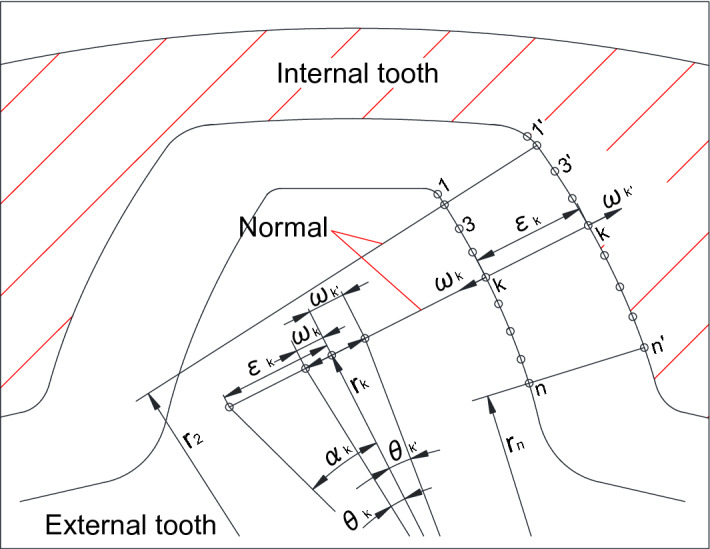
Concept diagram used for contact analysis of involute spline couplings.

Figure 2 is a concept diagram used to explain a new method presented by the author in this paper for loaded tooth contact analysis of a pair of involute spline couplings. In Fig. 2, one internal tooth space and one external tooth are illustrated. A lot of pairs of contact points, such as (1–1′), (2–2′), …, (k–k′), …, (n–n′), are made both on the internal and external tooth surfaces. These pairs of contact points are made along the directions of the common normal of the pairs of themselves. They are used as contact points in loaded tooth contact analysis of the spline couplings. The contact analysis of different pair of contact points is conducted along the different common normal of itself. Since the common normal at different place of the pair of contact points has different direction, loaded tooth contact analysis is conducted along different direction for different pair at the same time. This concept is completely different from the concept used in the papers^11–13^. This is a new concept presented firstly by the author in this paper. Though this new concept is presented to solve the contact problem of the spline couplings, it can also be suitable for other contact problems of elastic bodies with complex shapes.

In Fig. 2, (k–k′) is an arbitrary pair of contact points. For this pair of contact points, ${F}_{k}$ is a contact load between the pair (k–k′) along the common normal of this pair. ${\varepsilon }_{k}$ is a backlash between the pair (k–k′) along the common normal. ${\omega }_{k}$ and ${\omega }_{k {^{\prime}}}$ are deformation of the points k and k′ along the common normal. ${r}_{k}$ is a distance from the gear center to the common normal.

The linear deformation ${\omega }_{k}$ and ${\omega }_{k {^{\prime}}}$ can be converted to an angular deformation ${\theta }_{k}$ and ${\theta }_{k {^{\prime}}}$ by using Eq. (1). Also, the linear backlash ${\varepsilon }_{k}$ can be converted to an angular backlash ${\alpha }_{k}$ by using Eq. (2). The total angular deformation between the pair of contact teeth is denoted as $\alpha$.

1 $${\theta }_{k}={\omega }_{k}/{r}_{k}; {\theta }_{k {^{\prime}}}={\omega }_{k {^{\prime}}}/{r}_{k,}$$

2 $${\alpha }_{k}={\varepsilon }_{k}/{r}_{k}.$$

The loaded tooth contact analysis is conducted for a pair of spline couplings along the direction of rotation. As shown in Fig. 2, when the external gear is fixed and a torque is applied on the internal gear, if the pair of contact points (k–k′) comes into contact, then a relationship as given in Eq. (3) can be available and if (k–k′) does not come into contact, a relationship as given in Eq. (4) can be available. Equation (3) and Eq. (4) can be summarized into Eq. (5).

3 $${\theta }_{k}+{\theta }_{k {^{\prime}}}+{\alpha }_{k}-\alpha =0 \quad \left(k=1, 2, \dots , n\right) \quad \left(Contact\right),$$

4 $${\theta }_{k}+{\theta }_{k {^{\prime}}}+{\alpha }_{k}-\alpha >0 \quad  \left(k=1, 2, \dots , n\right) \quad \left(Not \; contact\right),$$

5 $${\theta }_{k}+{\theta }_{k {^{\prime}}}+{\alpha }_{k}-\alpha \ge 0 \quad  \left(k=1, 2, \dots , n\right).$$

Since ${\omega }_{k}$ and ${\omega }_{k {^{\prime}}}$ can be expressed using deformation influence coefficients ${a}_{kj}$ and ${a}_{k{^{\prime}}j {^{\prime}}}$ as given in Eqs. (6) and (7), Eq. (8) can be available by substituting Eqs. (6) and (7) into Eq. (1) and then substituting Eqs. (1) and (2) into Eq. (5). According to Eqs. (8), (9) can be available. Also, Eq. (9) can be rewritten into Eq. (10). If Eq. (10) is expressed with a form of matrix, Eq. (11) can be available. Variables of the matrices and arrays in Eq. (11) are given in Eqs. (12) to (15).

6 $${\omega }_{k}=\sum_{j=1}^{n}{a}_{kj}{F}_{j} \quad \left(k=1, 2, \dots , n\right),$$

7 $${\omega }_{k {^{\prime}}}=\sum_{j=1}^{n}{a}_{k {^{\prime}}j {^{\prime}}}{F}_{j} \quad \left(k=1, 2, \dots , n\right),$$

8 $$ \frac{1}{{r_{k} }}\mathop \sum \limits_{j = 1}^{n} a_{kj} F_{j} + \frac{1}{{r_{k} }}\mathop \sum \limits_{j = 1}^{n} a_{k^{\prime}j^{\prime}} F_{j} + \frac{{\varepsilon_{k} }}{{r_{k} }} - \alpha \ge 0 \quad \left( {k = 1, 2, \ldots , n} \right) , $$

9 $$ \mathop \sum \limits_{j = 1}^{n} \frac{1}{{r_{k} }}(a_{kj} + a_{k^{\prime}j^{\prime}} )F_{j} + \frac{{\varepsilon_{k} }}{{r_{k} }} - \alpha \ge 0 \quad \left( {k = 1, 2, \ldots , n} \right) , $$

10 $$ \mathop \sum \limits_{k = 1}^{n} \mathop \sum \limits_{j = 1}^{n} \frac{1}{{r_{k} }}(a_{kj} + a_{k^{\prime}j^{\prime}} )F_{j} + \mathop \sum \limits_{k = 1}^{n} \frac{{\varepsilon_{k} }}{{r_{k} }} - \mathop \sum \limits_{k = 1}^{n} \alpha \ge \mathop \sum \limits_{k = 1}^{n} 0, $$

11 $$ \left[ A \right]\left\{ F \right\} + \left\{ {\alpha_{k} } \right\} - \alpha \left\{ e \right\} \ge \left\{ 0 \right\}, $$

where,

12 $$\left[A\right]=\left[{A}_{kj}\right]=\left[\left({a}_{kj}+{a}_{k{^{\prime}}j{^{\prime}}}\right)/{r}_{k}\right],$$

13 $$\left\{F\right\}={\left\{{F}_{1}, {F}_{2}, \dots , {F}_{k},\dots ,{F}_{n} \right\}}^{T} ; {F}_{k}\ge 0 \left(k=1, 2, \dots , n\right),$$

14 $$\left\{{\alpha }_{k}\right\}={\left\{{\varepsilon }_{1}/{r}_{1}, {\varepsilon }_{2}/{r}_{2},\dots ,{\varepsilon }_{k}/{r}_{k},\dots ,{\varepsilon }_{n}/{r}_{n} \right\}}^{T},$$

15 $$\left\{0\right\}={\left\{0, 0, \dots , 0,\dots ,0 \right\}}^{T}.$$

On the other hand, there is a load equilibrium condition existing between contact loads of the contact points and the external torque $T$ that is applied on the contacted teeth. Here, $T={T}_{Z}/Z$. $Z$ is number of teeth. So, Eq. (16) can be available. If Eq. (16) is expressed with a form of matrix, Eq. (17) can be available.

16 $$\sum_{k=1}^{n}{F}_{k}\times {r}_{k}=T,$$

17 $${\left\{{r}_{k}\right\}}^{T}\left\{F\right\}=T.$$

Equation (11) is an inequality equation. It can be changed into an equality equation by introducing some positive variables (usually called slack variable in the Linear Programming Method) into Eq. (11). Then Eq. (11) can be changed into Eq. (18).

18 $$\left[A\right]\left\{F\right\}+\left\{{\alpha }_{k}\right\}-\alpha \left\{e\right\}-\left[I\right]\left\{Y\right\}=\left\{0\right\},$$

where, $\left[I\right]$ is a unit matrix and $\left\{Y\right\}$ are slack variables.

19 $$\left\{Y\right\}={\left\{{Y}_{1}, {Y}_{2}, \dots , {Y}_{k},\dots ,{Y}_{n} \right\}}^{T} ; {Y}_{k}\ge 0 \left(k=1, 2, \dots , n\right).$$

According to the principle of Linear Programming Method, an optimization model can be built as follows using the two equality Eqs. (17) and (18) through introducing an artificial variable $Z$. This is the mathematical model that is used to conduct contact analysis of the involute spline couplings in this paper.

#### Optimization model used for contact analysis of the involute spline couplings

##### Objective function

20 $$Z={X}_{n+1}+{X}_{n+2}+\dots +{X}_{n+n}+{X}_{n+n+1}.$$

##### Constraint conditions

21 $$-\left[A\right]\left\{F\right\}+\alpha \left\{e\right\}+\left[I\right]\left\{Y\right\}+\left[I\right]\left\{{Z}^{^{\prime}}\right\}=\left\{{\alpha }_{k}\right\},$$

22 $${\left\{{r}_{k}\right\}}^{T}\left\{F\right\}+{X}_{n+n+1}=T,$$

where,

23 $$\left\{Z{^{\prime}}\right\}={\left\{{X}_{n+1}, {X}_{n+2},\dots , {X}_{n+n}\right\}}^{T} ; {X}_{n+m}\ge 0 \quad \left(m=1, 2, \dots , n+1\right).$$

### FEM model of the spline couplings used for contact analysis

**Figure 3:**
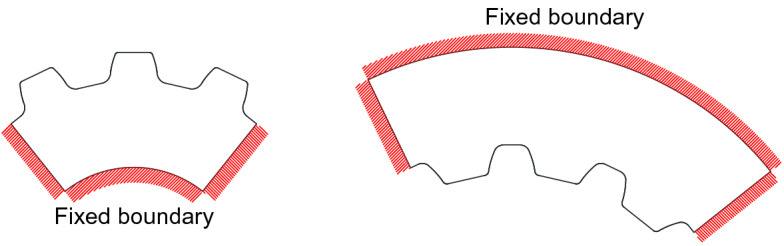
Section views of FEM models and boundary conditions.

3D, FEM are used to calculate the deformation influence coefficients ${a}_{kj}$ and ${a}_{k {^{\prime}}j {^{\prime}}}$ along the directions of the common normal of the pairs of contact points of themselves. Also stresses of the spline couplings are analyzed by the 3D, FEM. FEM boundary conditions used to calculate deformation influence coefficients and stresses are given in Fig. 3. For a pair of ideal spline couplings, since all the pairs of the contact teeth have the same contact and stress states, it is necessary only to consider one pair of teeth in the contact and stress analyses. So, this paper uses three-teeth models as shown in Fig. 3 for the contact and stress analyses in order to be able to calculate the deformation influence coefficients precisely. But the contact and stress analyses are only conducted for the middle pair of teeth. In Fig. 3, the hatched boundaries are fixed as FEM boundary conditions.

**Figure 4:**
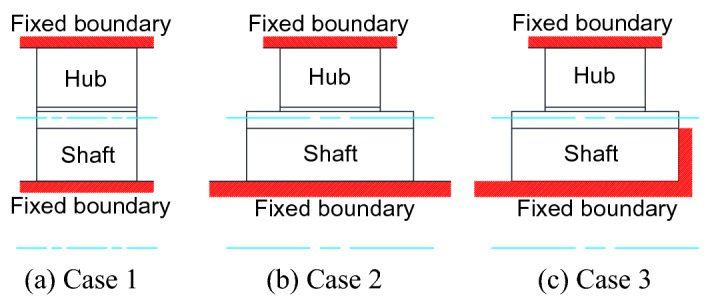
Three cases used for FEM boundary conditions in the contact analysis.

The shaft and hub splines are fixed along axial directions under three cases (Cases 1, 2 and 3) as shown in Fig. 4. Case 1 is the one that both the external and internal teeth have the same face widths and axial boundaries of the shaft and hub splines are fixed as shown in Fig. 4a. Case 2 is the one that the external and internal teeth have different face widths and the axial boundaries are fixed as shown in Fig. 4b. Case 3 is the one that the external and internal teeth have different face widths and the axial boundaries are fixed as shown in Fig. 4c. In Fig. 4c, one end face is added in the fixed boundaries by comparing Fig. 4c with Fig. 4b in order to investigate the effect of the end face on tooth contact and stress distribution states.

### Flowchart of software development

**Figure 5:**
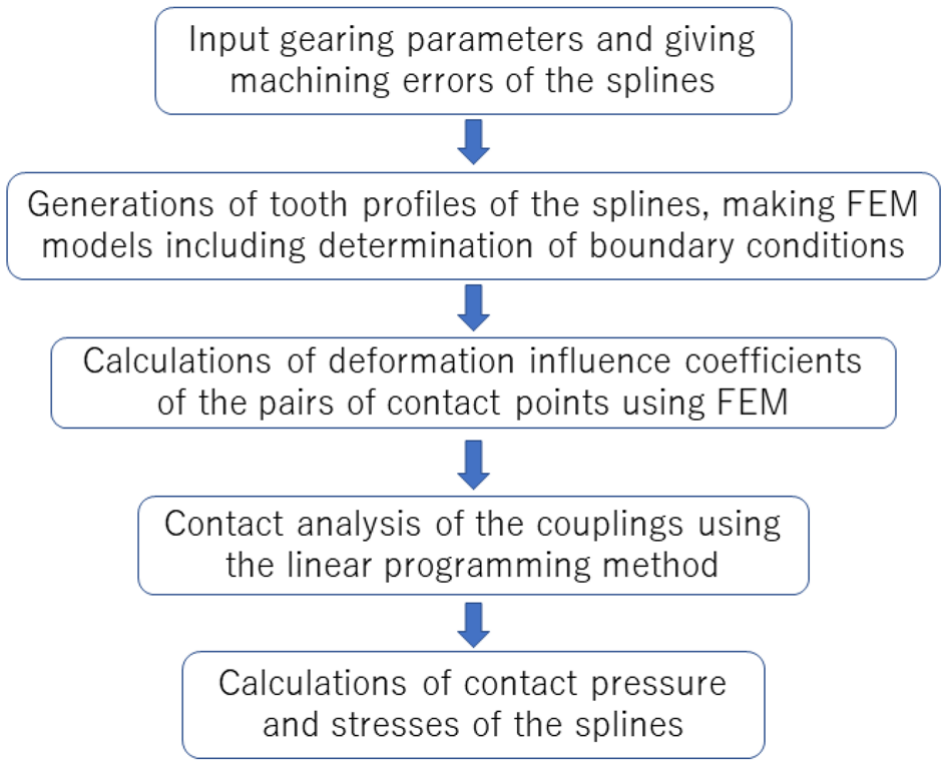
Flowchart of software development.

Special FEM software is developed for contact and stress analyses of the involute spline couplings based on the principle and optimization model stated above using Linear Programming Method and FEM. The flowchart for the software development is given in Fig. 5. With the help of the developed software, the contact and stress analyses are conducted for the pair of spline couplings as given in Table 1. Calculation results are introduced in “Contact and stress analyses of the couplings with the same face widths”, “Contact and stress analyses of the couplings with different face widths” and “The Effect of machining errors on stress distributions of the couplings”.

## Contact and stress analyses of the couplings with the same face widths

### Generations of tooth profiles and FEM models of the spline couplings

**Figure 6:**
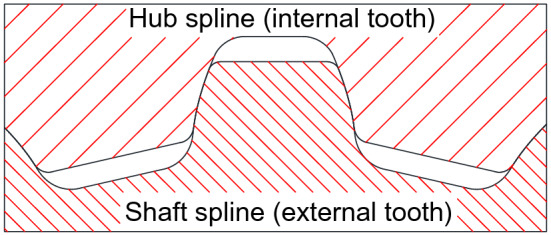
Generations of external and internal tooth profiles of the couplings.

Software used to generate tooth profiles of the splines is developed specially by the author. When the gearing parameters as shown in Table 1 are inputted into the software, tooth profiles of both the external and internal teeth can be generated automatically. Figure 6 is the result of tooth profiles of the splines generated by the developed software. The generated tooth profiles are used to build FEM models of the couplings automatically by other software developed by author.

**Figure 7:**
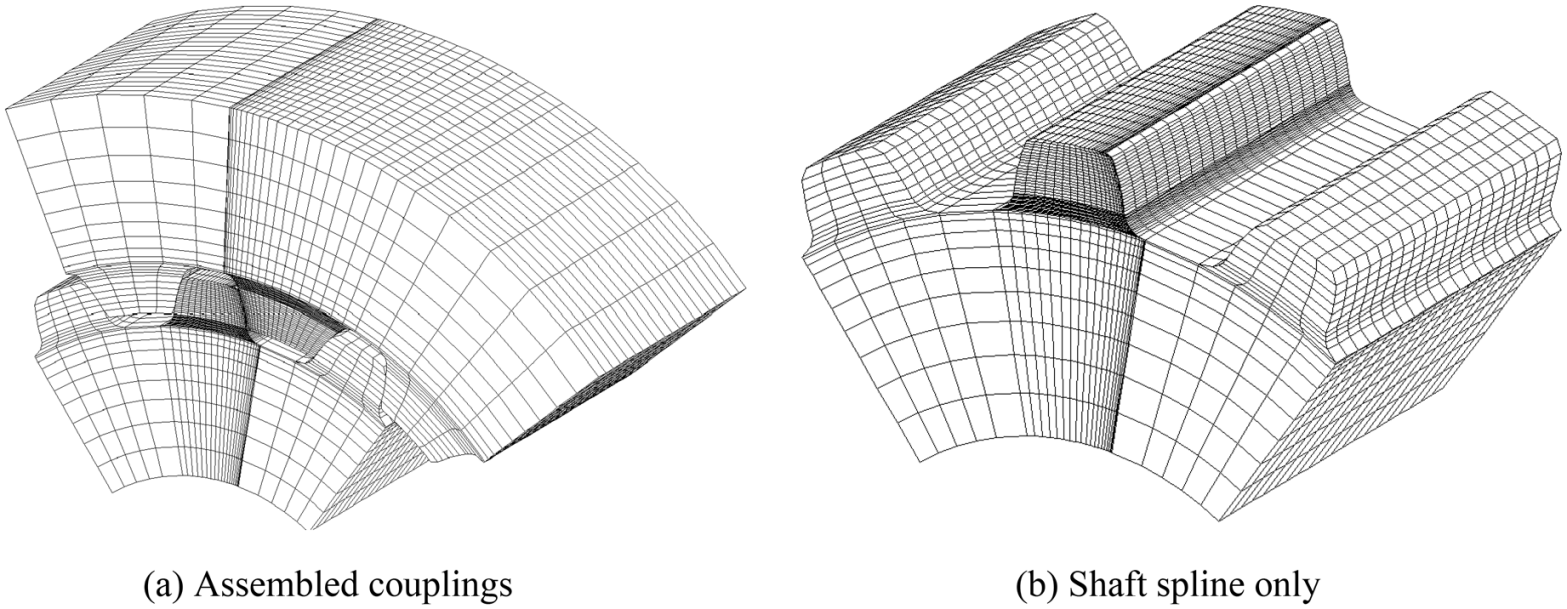
FEM model of the involute spline couplings with the same face widths.

Figure 7a is a FEM model of assembled spline couplings with the same face widths as shown in Fig. 1b. Figure 7b is the FEM model of the shaft spline only. Since the contact and stress analyses are only conducted for the middle pair of teeth, FEM meshes of the middle pair of teeth are divided to be very fine. As shown in Fig. 7, meshes near to the contact surfaces of the middle pair of teeth and meshes at the tooth roots of the middle pair of teeth are divided to be very fine to obtain reliable calculation results. Contact and stress analyses are conducted for the couplings under the boundary condition of Case 1 as shown in Fig. 4a.

### Calculation results obtained by the developed FEM software

**Figure 8:**
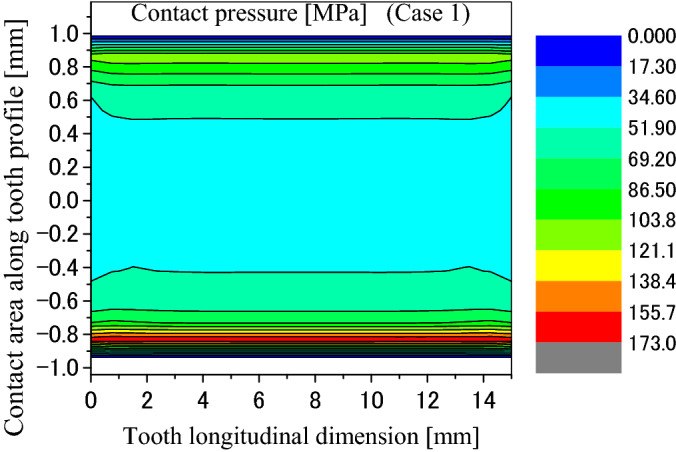
Contour lines of contact pressure distribution on the tooth surface (Case 1).

**Figure 9:**
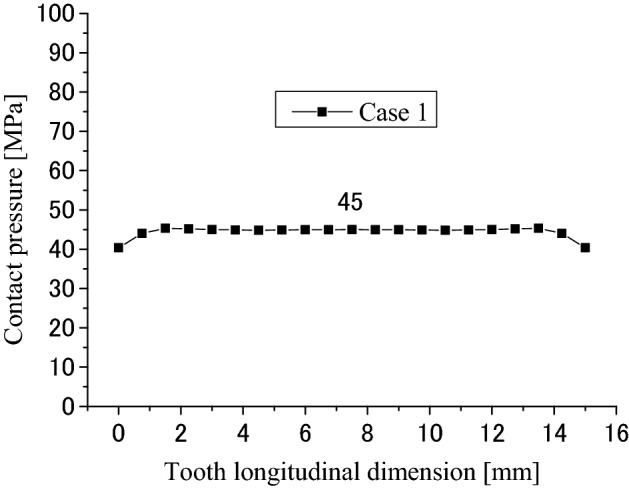
Contact pressure distribution along tooth longitude (Case 1).

**Figure 10:**
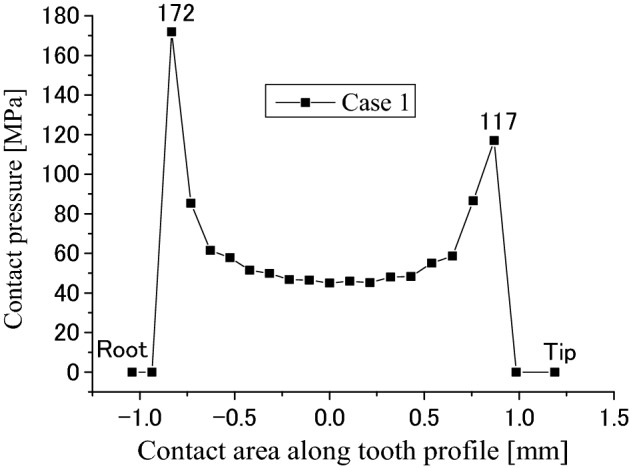
Contact pressure distribution along tooth profile (Case 1).

Firstly, calculation results obtained by the developed FEM software are introduced in this section. Figure 8 is a pressure distribution on contacted surface of the external tooth. In Fig. 8, the abscissa is a longitudinal dimension of contact points along the lead. “$x=0$” stands for the left end of the tooth as shown in Fig. 1b. The ordinate of Fig. 8 is a dimension of the contact point along the tooth profile. “$y=1$” stands for the root of the internal tooth and the tip of the external tooth. “$y=-1$” stands for the tip of the internal tooth and the root of the external tooth. Figures 9 and 10 are 2D, distributions of the contact pressure along the lead and tooth profile respectively. They are results at the tooth center positions. From Fig. 9, it is found that the contact pressure is almost a uniform distribution along the lead. The maximum contact pressure is about 45 MPa, a very low pressure. From Fig. 10, it is found that the contact pressure is varied greatly along the tooth profile. Greater contact pressures occurred at tooth tip and root contacts. This phenomenon is often called “Edge load”. The maximum pressure is about 172 MPa at the root of the external tooth. Though this value is much greater than 45 MPa, it is still a very low contact pressure by comparing it with the allowable contact pressure of gears. So, it can be said that it is rare for the involute spline couplings to have contact fatigue failure of the teeth. If it does happen, the root and tip shall be the positions to have the failures.

**Figure 11:**
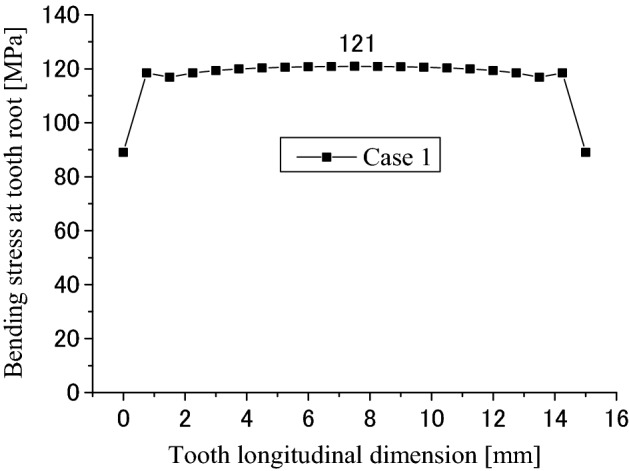
Root bending stress distribution along tooth longitude (Case 1).

Figure 11 is tooth bending stress distribution at the root along the lead. The abscissa is the tooth longitudinal dimension and the ordinate is the bending stress at the tensile side of tooth root. It is found that the tensile stress is almost a uniform distribution along the lead and the maximum tensile stress is about 121 MPa. This value is about 1/3 of the allowable stress of a pair of gears. So, it can be said that it is also rare for the spline couplings to have bending fatigue failure at the root.

**Figure 12:**
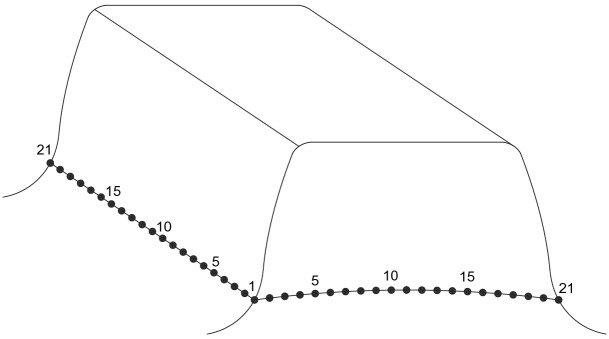
Nodal numbers along the circumference and the longitude of the external tooth.

**Figure 13:**
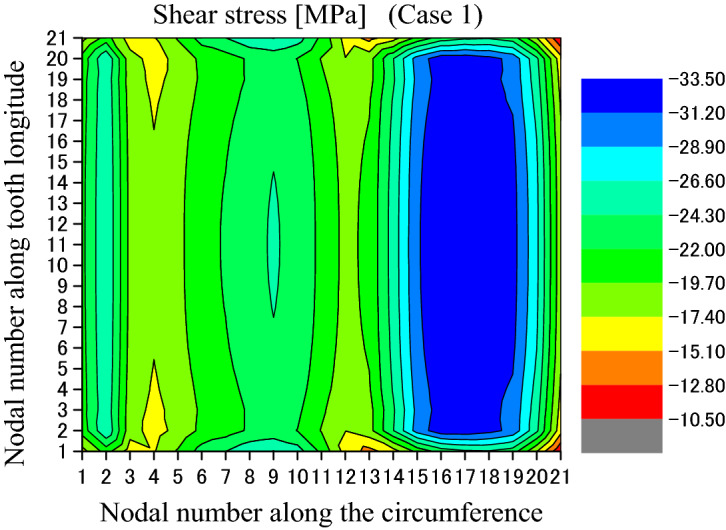
Shear stress distributed on the circumferential surface (Case 1).

**Figure 14:**
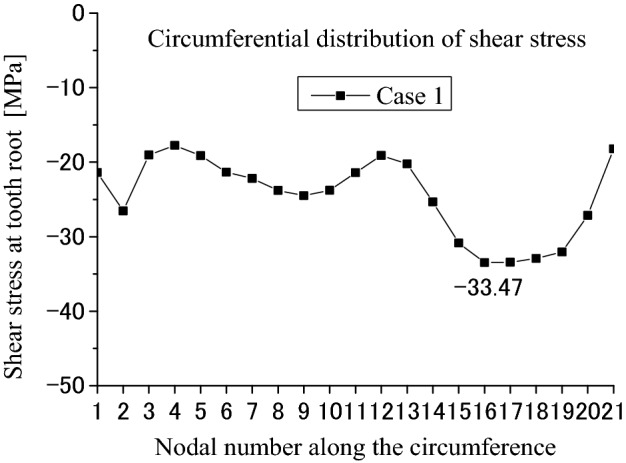
Circumferential distribution of the shear stress (Case 1).

Figure 12 is used to show some number positions on a circumferential surface at the tooth bottom of the shaft spline where there is the maximum shear stress existing on this surface. In Fig. 12, nodal numbers (1, 2, 3, …, 21) along the circumferential direction and the nodal numbers (1, 2, 3, …, 21) along tooth longitude are illustrated. These numbers are used in Figs. 13 and 14. Figure 13 is a shear stress distribution on this circumferential surface. In Fig. 13, the abscissa is the nodal numbers (1, 2, 3, …, 21) along the circumferential direction and the ordinate is the nodal numbers (1, 2, 3, …, 21) along tooth longitude. From Fig. 13, it is found that there is greater shear stress existing in the bule aera. Figure 14 is a 2D, distribution of the shear stress along the circumferential direction. It is the shear stress of the middle node of the tooth longitude. So, the abscissa of Fig. 14 is the nodal numbers (1, 2, 3, …, 21) along the circumferential direction and the ordinate is the shear stress at the middle node along the lead. From Fig. 14, it is found that the maximum shear stress (− 33.47 MPa) exists at the nodal number 16. Since this value is very close to the allowable shear stress (for an example, 40 MPa for S45C, a Japanese material), the involute spline couplings shall have shear fatigue failure very easily.

### Calculation results obtained by an approximation method

**Figure 15:**
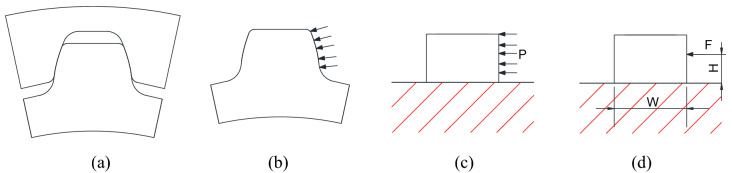
Simplified models of the involute spline couplings.

Stresses are also calculated using an approximation method that is often used by engineers in machine design. Figure 15a–d are concept diagrams used to explain the approximation method. Figure 15a is an image that the shaft spline contacts with the hub spline. Figure 15b is an image that contact loads are applied on the tooth surface after the hub spline is removed. Since the tooth profile is involute and the load distribution along the profile is not a uniform, it is difficult to calculate the contact pressure on tooth surface, the bending and shear stresses at the tooth root precisely using a theoretical method. So, the model in Fig. 15b is simplified into a simple model as shown in Fig. 15c. In Fig. 15c, the involute curve is simplified into a straight line and the ununiform tooth load distribution is simplified into a uniform distribution. Under the help of these simplifications, contact pressure distribution on tooth surface, bending and shear stresses at the tooth root can be calculated approximately. Figure 15c is used to calculate pressure distribution on the tooth surface approximately. This model is furthermore simplified into Fig. 15d in order to calculate the bending and shear stresses at the tooth root. In Fig. 15d, the distribution load in Fig. 15c is simplified into a concentrated load applied at the middle point of the tooth working depth. Then, the bending and shear stresses at the root can be calculated as a cantilever using the formulas introduced in the mechanics of material.

The contact pressure, bending stresses and shear stresses are calculated as follows using the approximation method. If the contact pressure on tooth surface is denoted as $P$, the total load on one pair of teeth is denoted as $F$, working depth of the pair of teeth is denoted as $h$, face width of teeth is denoted as $b$, number of teeth is denoted as $Z$, diameter of the pitch circle is denoted as ${D}_{m}$, then $F$ and $P$ can be calculated approximately by Eqs. (24) and (25). Shear stress $\tau$ at the tooth root can be calculated approximately by Eq. (26). In Eq. (26), $\mathrm{W}$ is tooth thickness at the root as shown in Fig. 15d. The bending stress $\sigma$ at the tooth root can be calculated approximately by Eq. (27). In Eq. (27), $H$ is a distance from the middle point of the tooth working depth to the tooth root as shown in Fig. 15d.

24 $$F=T/(0.5{D}_{m}Z),$$

25 $$P=F/(h\times b),$$

26 $$\tau =F/(bW),$$

27 $$\sigma =6FH/(b{W}^{2}).$$

### Comparison between the FEM software and the approximation method

Calculation results are compared between the developed FEM software and the approximation method in Table 2. In Table 2, it is found that tooth contact stress obtained by the FEM software is about 3.4 times greater than the one obtained by the approximation method. The shear stress obtained by the FEM software is about 1.3 times greater than the one obtained by the approximation method. The bending stress obtained by the FEM software is about 1.9 times greater than the one obtained by the approximation method. So, it can be said that the approximation method is not precise enough for strength calculations of the involute spline couplings in machine design.

**Table 2:** Comparison between the FEM software and the approximation method.

The maximum stresses	Developed FEM software	Approximation method
Tooth contact stress (MPa)	172	50.3
Shear stress at tooth root (MPa)	33.5	25.6
Bending stress at tooth root (MPa)	121	63.5

## Contact and stress analyses of the couplings with different face widths

### FEM model and boundary conditions

**Figure 16:**
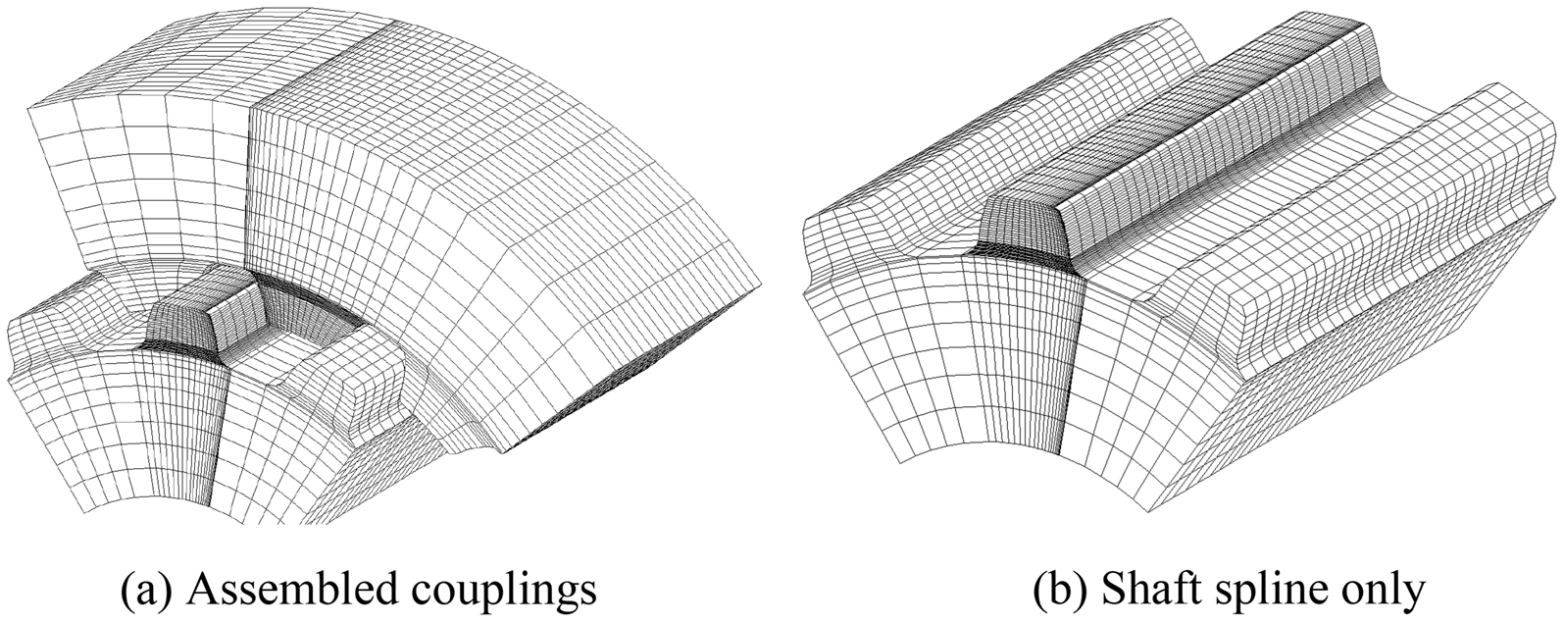
FEM model of the involute spline couplings with different face widths.

Contact and stress analyses are also conducted for the spline couplings with difference face widths. Figure 16a is a FEM model of assembled spline couplings with different face widths as shown in Fig. 1c. Figure 16b is the FEM model of the shaft spline only. From Fig. 16, it can be found that the face width of the shaft spline is much wider than that of the hub spline. FEM meshes are divided to be very fine for the middle pair of teeth.

### Strength analysis using the boundary condition of Case 2 as shown in Fig. 4b

**Figure 17:**
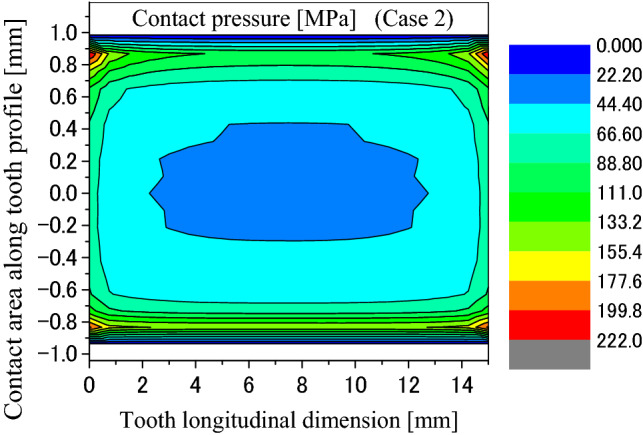
Contour lines of contact pressure distribution on tooth surface (Case 2).

**Figure 18:**
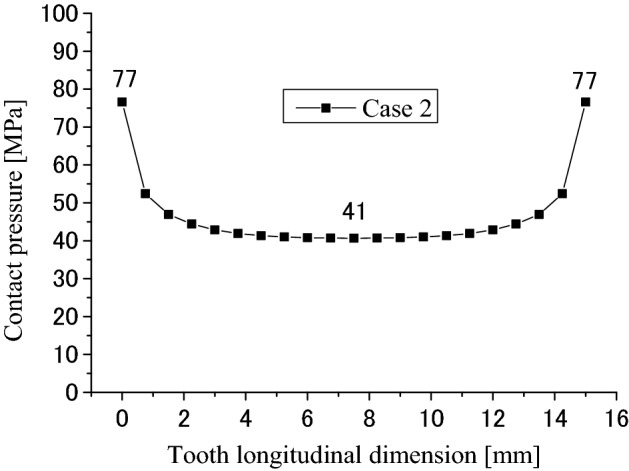
Contact pressure distribution along tooth longitude (Case 2).

**Figure 19:**
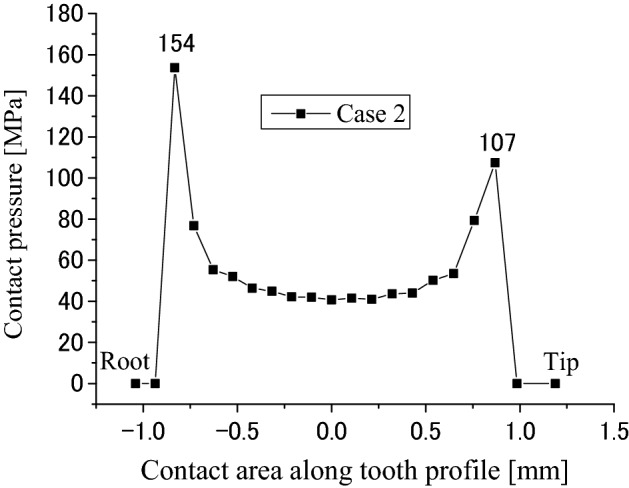
Contact pressure distribution along tooth profile (Case 2).

Contact and stress analyses are conducted for the couplings under the boundary condition of Case 2 as shown in Fig. 4b. Figure 17 is a pressure distribution on the middle pair of contacted teeth. The abscissa and the ordinate are the same as Fig. 8 respectively. Figures 18 and 19 are 2D, distributions of the contact pressure. Also, the abscissa and the ordinate are the same as Figs. 9 and 10 respectively. From Fig. 18, it is found that the contact pressure is not a uniform distribution along the lead. “Edge loads” exist at the two ends of the face width because of the tooth contact with different face widths. The maximum contact pressure is about 77 MPa. This value is also very low for the spline couplings. From Fig. 19, it is found that the contact pressure distribution is varied very great along the tooth profile. “Edge loads” exist at the tooth tip and root contacts. The maximum contact pressure is about 154 MPa. This value is a little lower than 172 MPa that is introduced in Fig. 10. This means that the tooth contact with different face widths reduced the “edge loads” a little. Since 154 MPa is much lower than the allowable pressure of gears (for an example, 1400 MPa), it can be said that the tooth contact fatigue failure of the spline couplings shall not happen.

**Figure 20:**
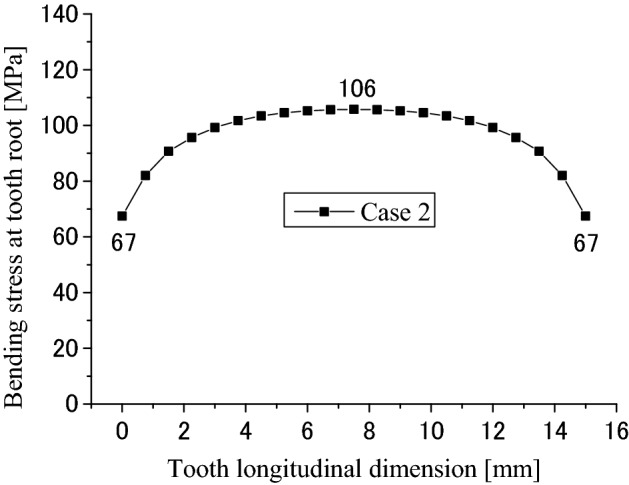
Root bending stress distribution along tooth longitude (Case 2).

Figure 20 is the root bending stress distribution along the lead. The abscissa is the longitudinal dimension of the contacted teeth and the ordinate is the bending stress at the tensile side of the contacted teeth. It is found that the bending stress is not a uniform distribution along the lead. The maximum stress at the center of the face width is about 106 MPa. This value is a litter lower than the value 121 MPa in Fig. 11. This means that the tooth contact with different face widths also reduced the maximum bending stress of the tooth root.

**Figure 21:**
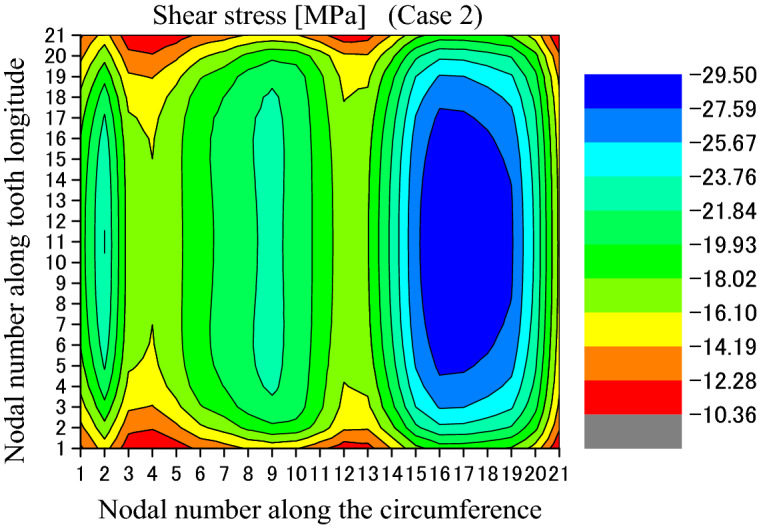
Shear stress distributed on the circumferential surface (Case 2).

**Figure 22:**
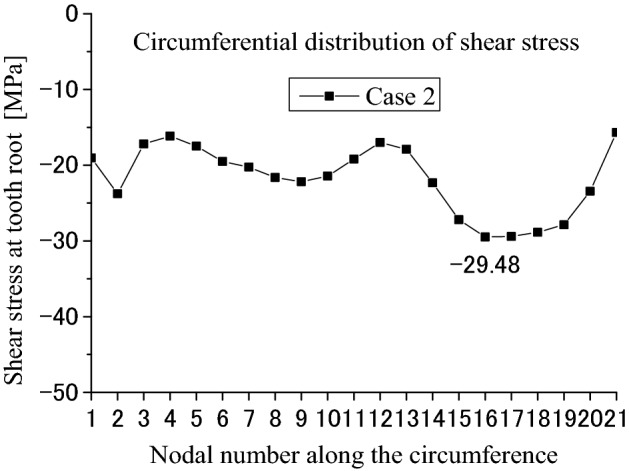
Circumferential distribution of the shear stress (Case 2).

Figure 21 is the shear stress distributed on the circumferential surface as shown in Fig. 12. In Fig. 21, the abscissa and the ordinate are the same as Fig. 13. From Fig. 21, it is found that the shear stress distribution has a little change because of the different face widths. Figure 22 is a 2D, distribution of the shear stress along the circumferential direction. The abscissa and the ordinate are the same as Fig. 14. From Fig. 22, it is found that the maximum shear stress is − 29.48 MPa. This value is a little lower than − 33.47 MPa given in Fig. 14 because of the different face widths. This means that the different face widths can also reduce the maximum shear stress a little.

### Strength analysis using the boundary condition of Case 3 as shown in Fig. 4c

**Figure 23:**
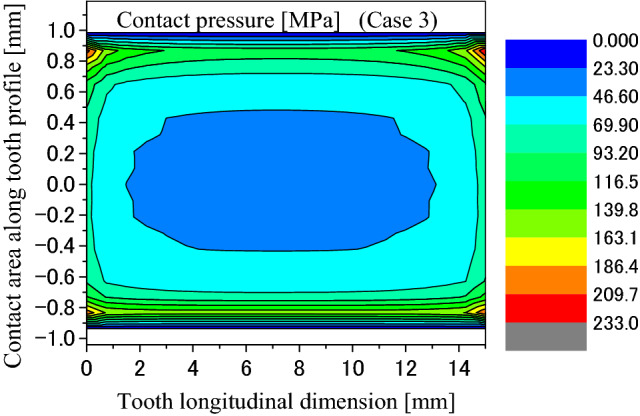
Contour lines of contact pressure distribution on tooth surface (Case 3).

**Figure 24:**
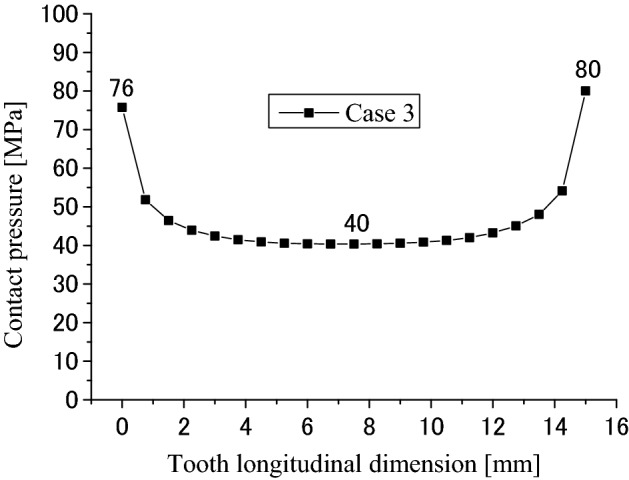
Contact pressure distribution along tooth longitude (Case 3).

**Figure 25:**
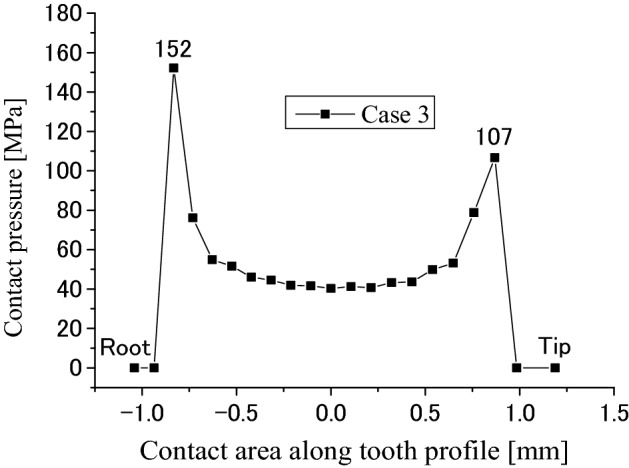
Contact pressure distribution along tooth profile (Case 3).

**Figure 26:**
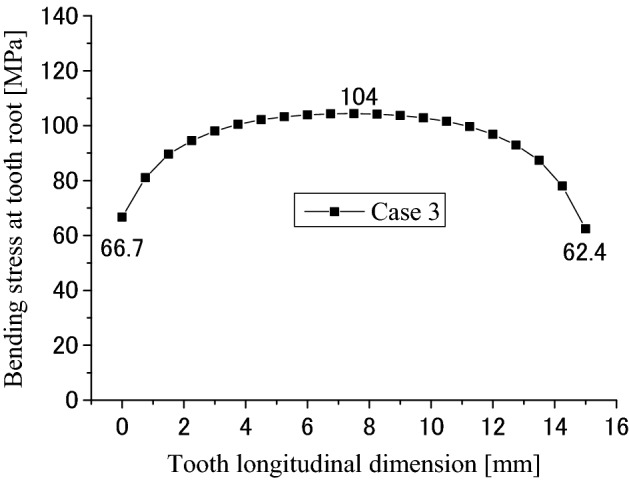
Longitudinal distribution of the root bending stress (Case 3).

**Figure 27:**
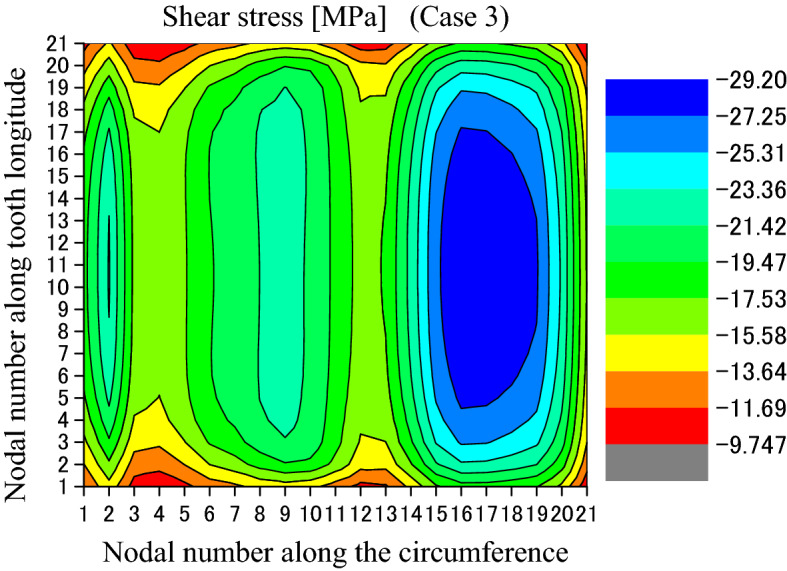
Shear stress distributed on the circumferential surface (Case 3).

**Figure 28:**
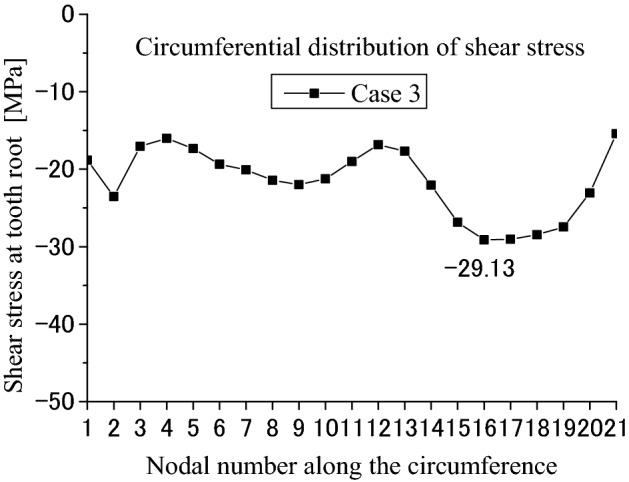
Circumferential distribution of the shear stress (Case 3).

Contact and stress analyses are conducted for the couplings under the boundary condition of Case 3 as shown in Fig. 4c to investigate the effect of different boundary conditions on stress distributions. Calculation results are given in Figs. 23, 24, 25, 26, 27 and 28. By comparing these results with Figs. 17, 18, 19, 20, 21 and 22, it is found that there is very little difference between the boundary conditions Case 2 and Case 3. This means that both the two boundary conditions as shown in Fig. 4b,c can be used for contact and stress analyses of the spline couplings.

## The effect of machining errors on stress distributions of the couplings

**Figure 29:**
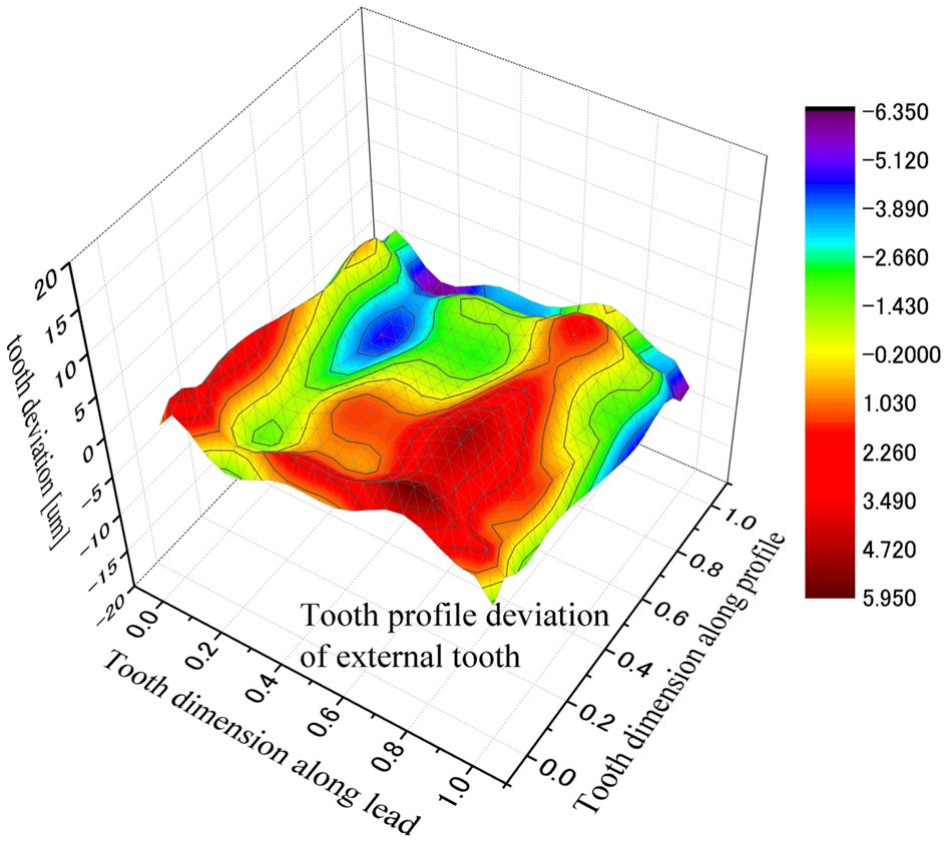
3D, tooth profile deviations of an external tooth.

**Figure 30:**
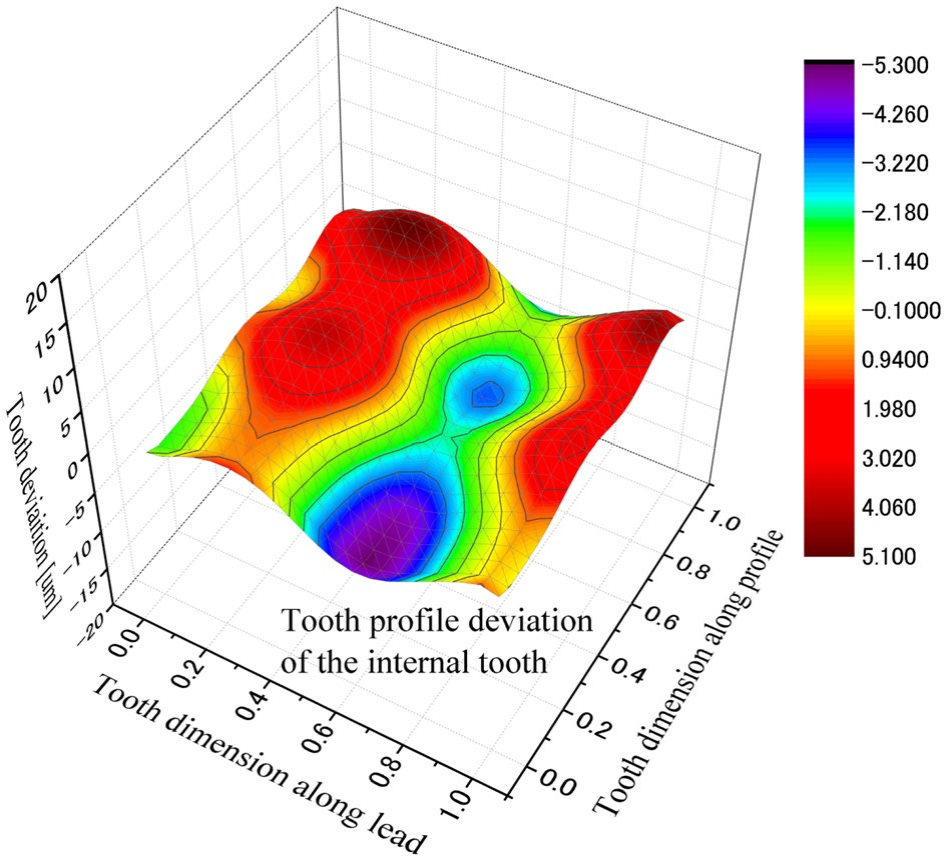
3D, tooth profile deviations of an internal tooth.

Effect of tooth machining errors on coupling strength is investigated in the paper. Figure 29 is a 3D, distribution of tooth profile deviations of the external tooth. Figure 30 is a 3D, distribution of tooth profile deviations of the internal tooth. These profile deviations are used in contact and stress analyses of the couplings under the same calculation and boundary conditions of Case 1.

**Figure 31:**
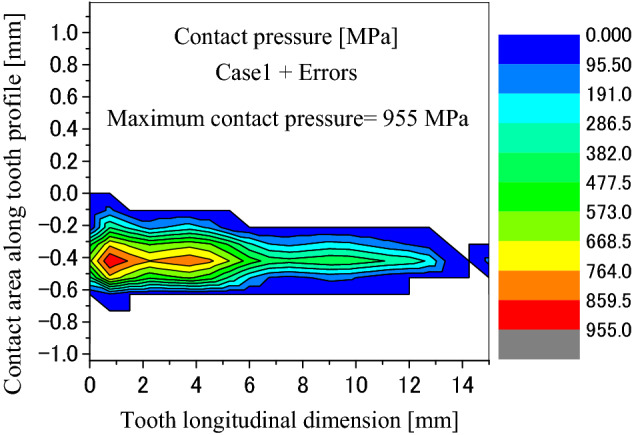
Contour lines of contact pressure distribution on tooth surface (Case 1 + Errors).

Figure 31 is the tooth contact pressure distribution calculated under tooth profile deviations. It is found that the contact pressure distribution is changed very much because of the tooth profile deviations by comparing Fig. 31 with Fig. 8. Tooth contact pattern is changed from a full tooth contact into a partial tooth contact. Also, the maximum contact pressure is changed from 172 into 955 MPa. This is a very great pressure that is possible enough to result in tooth contact fatigue failures.

**Figure 32:**
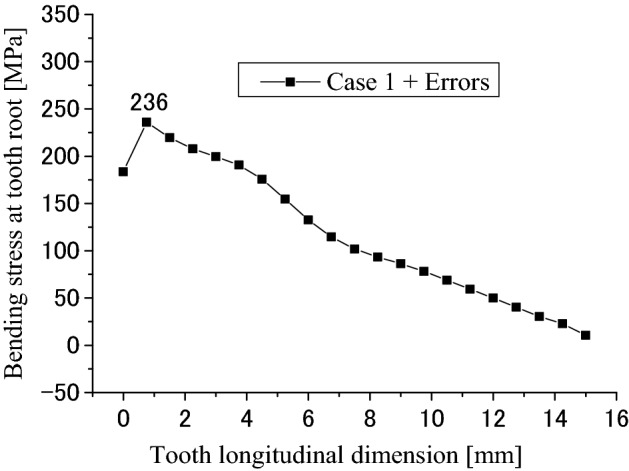
Longitudinal distribution of the root bending stress (Case 1 + Errors).

Figure 32 is the tooth bending stress distribution when the profile deviations are considered. It is found that the bending stress distribution has also a great change because of the profile deviations by comparing Fig. 32 with Fig. 11. The bending stress distribution is changed from a uniform distribution into an inclined distribution. The maximum bending stress is changed from 121 into 236 MPa. This value is also a great stress that maybe results in the tooth bending fatigue failure.

**Figure 33:**
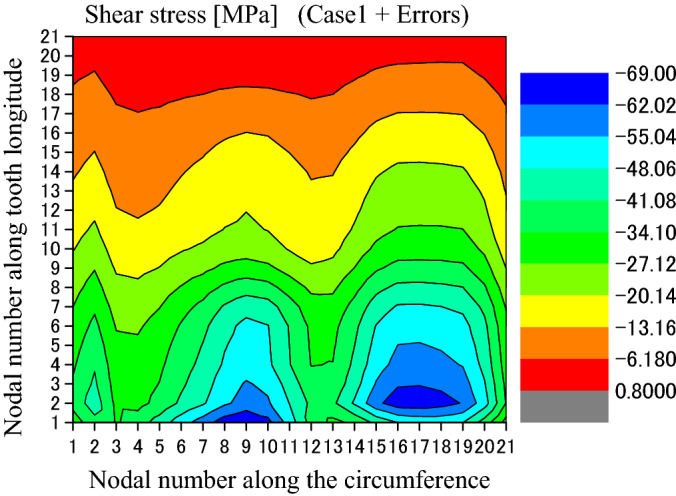
Shear stress distributed on the circumferential surface (Case 1 + Errors).

Figure 33 is the shear stress distribution on the circumferential surface as shown in Fig. 12 when the tooth profile deviations are considered. It is found that the shear stress distribution has also a great change because of the profile deviations by comparing Fig. 33 with Fig. 13. The maximum shear stress is changed from 33.5 into 69 MPa. This value exceeds the allowable shear stress (for an example, 40 MPa for S45C, a Japanese material). So, the shear fatigue failure at the tooth root shall be the most dangerous failure pattern for the involute spline couplings.

## Effects of pitch errors on tooth contact pressure distributions of the couplings

Effects of pitch errors of the spline teeth on tooth contact pressure distributions are investigated here. To be able to realize this investigation, a loaded tooth contact analysis must be conducted for all the pairs of contact teeth of the spline couplings. So, Eq. (10) is extended into Eqs. (28) and (16) is extended into Eq. (29). In Eqs. (28) and (29), $z$ is the number of teeth of the spline couplings. $z$ is equal to 14 according to Table 1. Also, ${\alpha }_{z}$ is the total angular deformation of the shaft spline relative to the hub spline. ${T}_{Z}$ is the total external torque of the couplings. ${T}_{Z}$ is 411.6 Nm according to Table 1.

28 $$ \mathop \sum \limits_{k = 1}^{n \times z} \mathop \sum \limits_{j = 1}^{n \times z} \frac{1}{{r_{k} }}(a_{kj} + a_{k^{\prime}j^{\prime}} )F_{j} + \mathop \sum \limits_{k = 1}^{n \times z} \frac{{\varepsilon_{k} }}{{r_{k} }} - \mathop \sum \limits_{k = 1}^{n \times z} \alpha_{z} \ge \mathop \sum \limits_{k = 1}^{n \times z} 0 , $$

29 $$ \mathop \sum \limits_{k = 1}^{n \times z} F_{k} \times r_{k} = T_{Z} \left( {k = 1,2,3, \ldots n; z = 1,2,3, \ldots 14} \right). $$

A new mathematical model used to solve the problem of all the pairs of contact teeth can also be built up like Eqs. (20) to (23) based on Eqs. (28) and (29). To save space of the paper, this new model is omitted here. In the new model, pitch errors of every pair of contact teeth can be considered into ${\varepsilon }_{k}$ of every pair of contact points. Then the contact problem of all-tooth-contact model is solved with the same method used for the contact problem of one-tooth-contact model as given in Eqs. (20) to (23). Calculation results are introduced in the following.

**Figure 34:**
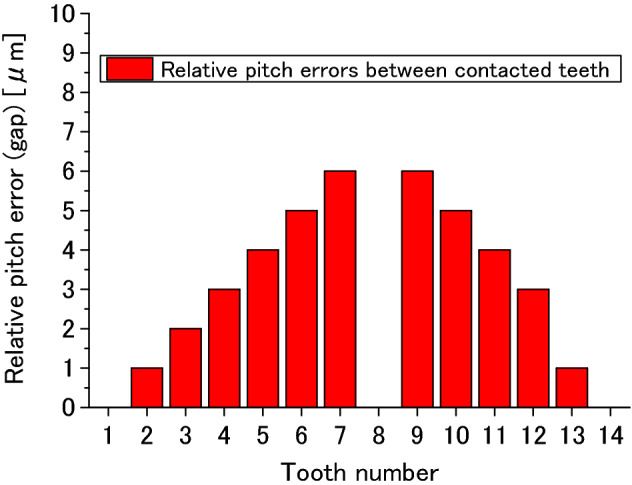
Relative pitch errors of every pair of contact teeth.

To investigate the effect of pitch errors on tooth contact, pitch errors are given to all the pairs of contact teeth at the first. Then, gaps of all the pairs of contacted teeth are calculated according to the pitch errors. This gap is called “relative pitch error” here. Figure 34 is the relative pitch error of every pair of contact teeth. In Fig. 34, the abscissa is tooth number and the ordinate is the relative pitch error. 14 pairs of contact teeth are used in the contact analysis. From Fig. 34, it is found that relative pitch errors of Tooth 1, Tooth 8 and Tooth 14 are zero while Tooth 7 and Tooth 9 have the maximum values of 6 mikron. These relative pitch errors in Fig. 34 are used in the contact analysis of all the pairs of teeth. Some results obtained by this analysis are introduced in the following.

**Figure 35:**
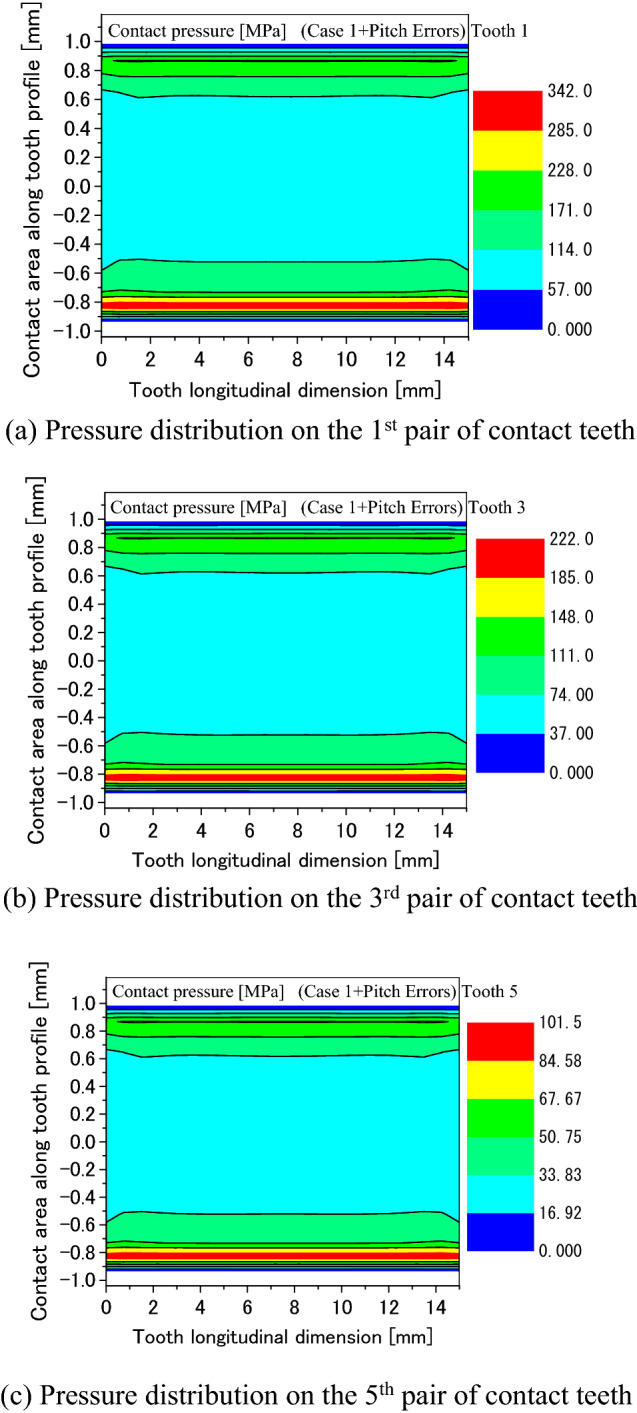
Effect of the pitch errors on tooth pressure distribution.

Figure 35a–c are calculated contact pressure distributions on the surfaces of Tooth 1, Tooth 3 and Tooth 5. In Fig. 35, it is found that the maximum contact pressure is getting smaller as the relative pitch error (gap) increases. This means that the pitch errors have great effects on tooth contact pressure distributions of spline couplings. It is also found that the contact pressures on Tooth 7 and Tooth 8 are calculated to be zero. This is because the relative pitch errors of these two pairs of teeth are 6 mikron, the maximum gaps among all the pairs of contact teeth. Comparing Fig. 8 to Fig. 35a, it is found that the maximum contact pressure is about 173 MPa in Fig. 8 while the maximum contact pressure is about 342 MPa in Fig. 35a. This value is about twice of the 173 MPa. So, it is necessary to consider the pitch errors in strength calculations of the spline couplings.

## Reliability of the method and developed software

Since there is not an effective method available at the present situation to measure pressure distributions between the contact teeth, it is quite a difficult thing to verify reliability of the methods and software presented in this paper experimentally. So, it is verified indirectly in theory here.

**Figure 36:**
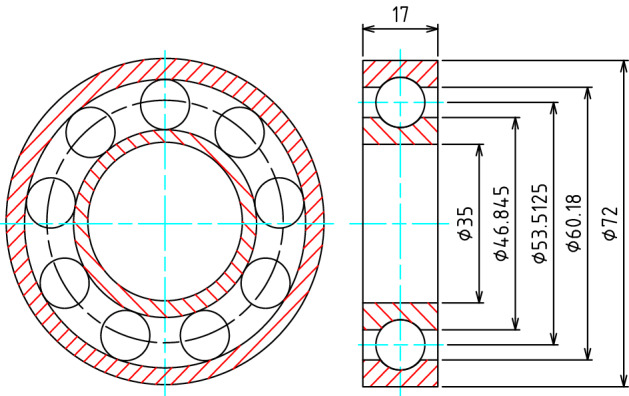
Structure and dimension of the ball bearing.

**Figure 37:**
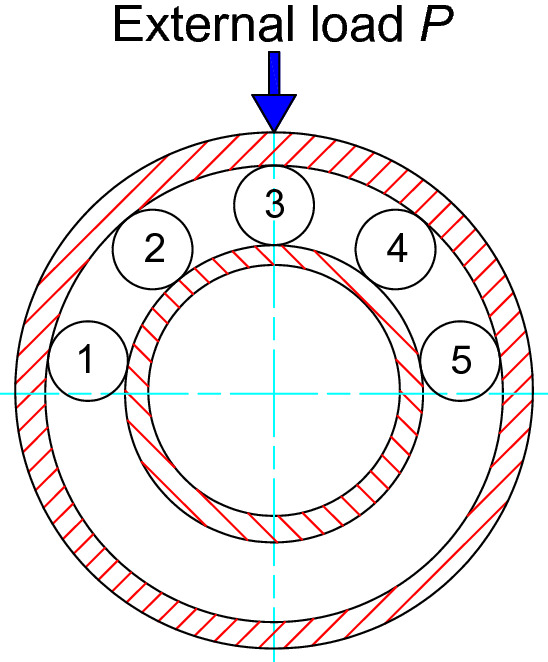
Structure and dimension of the ball bearing.

The developed methods and software are used to analyze contact stress and radial deformation of a ball bearing (type number 6332) numerically. Also, the same analyses are conducted by using commercial software Abaqus^14^ three-dimensionally and Hertz’s formula. Figure 36 is structure and dimension of the ball bearing used as the research object. Figure 37 is an image to do the contact stress and radial deformation of the ball bearing. As shown in Fig. 37, the inner circumferential surface of the inner ring is fixed as the boundary condition of the finite element analysis. An external load *P* is applied on the outer circumferential surface of outer ring radially. The quadratic tetrahedral element is used in the analysis.

**Figure 38:**
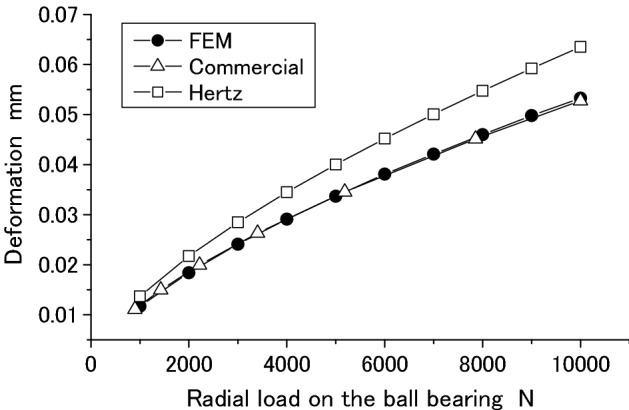
Radial deformation of a ball bearing.

**Figure 39:**
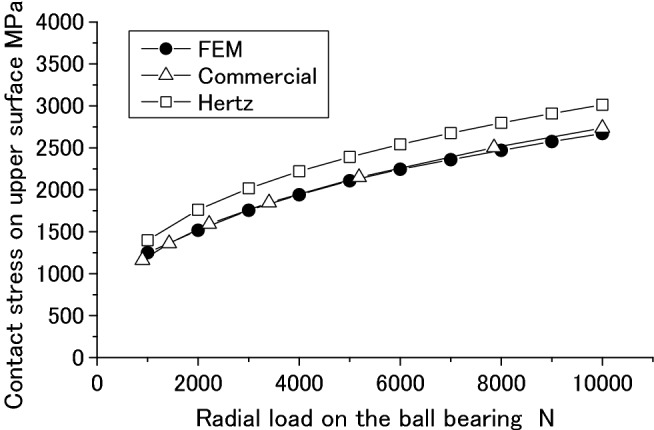
The maximum contact stress on the ball.

Figures 38 and 39 are comparisons of the radial deformation and contact stress of the ball bearing respectively among the three methods. In Figs. 38 and 39, “FEM” is used to represent the results computed by the methods and software developed in this paper. “Commercial” and “Hertz” are used to represent the results computed by the Commercial software and Hertz’s formula respectively. From Figs. 38 and 39, it is found that the radial deformation and the maximum contact stress calculated by the methods presented in this paper are almost the same as the results obtained by the Commercial software. This means that the methods and software presented in this paper are reliable.

## Conclusions

A numerical method is presented in this paper to conduct loaded tooth contact analysis and stress calculations of involute spline couplings. Special FEM software has been developed successfully.

Contact stresses on the tooth surface, bending and shear stresses at the tooth root are analyzed successfully for a pair of ideal involute spline couplings without machining and assembly errors. It is found that edge loads exist at the tooth tip and root contacts. It is also found that the maximum contact stress on the tooth surface and the maximum bending stress at the tooth root are so small that they cannot result in the tooth surface and root fatigue failures. Since the maximum shear stress at the tooth root is very close to the allowable stress, it is possible enough for the pair of ideal involute spline couplings to have shear fatigue failure at the tooth root.

Contact and stress analyses are also conducted for a pair of real involute spline couplings with tooth profile deviations to investigate the effect of the tooth profile deviations on stress distributions of the spline couplings. It is found that the contact stresses, bending and shear stresses become very great because of the tooth profile deviations. Also, the shear stress exceeds the allowable stress because of the tooth profile deviations. So, it is possible enough for the spline couplings to have the contact fatigue failure on tooth surface, bending and shear fatigue failures at the tooth root if the machining errors of the teeth are considered.

It is suggested to consider the effect of the tooth machining errors in strength calculations of the involute spline couplings when to design the spline couplings.

## Data Availability

The datasets used and/or analyzed during the current study available from the corresponding author on reasonable request.
